# Longitudinal Model Identification and Controller Design for a Fish Robot with Control Fins via Experiments

**DOI:** 10.3390/biomimetics10110731

**Published:** 2025-11-01

**Authors:** Daewook Kim, Jinyou Kim, Changyong Oh, Taesam Kang

**Affiliations:** 1 Department of Aerospace Information Engineering, Konkuk University, Seoul 05029, Republic of Korea; eodnr1000@konkuk.ac.kr (D.K.); ocy4262@konkuk.ac.kr (C.O.); 2 Department of Smart Vehicle Engineering, Konkuk University, Seoul 05029, Republic of Korea; qocnrlacl9@konkuk.ac.kr; 3 Department of Aerospace-Mobility Engineering, Konkuk University, Seoul 05029, Republic of Korea; 4 Future Drone Center, Konkuk University, Seoul 05029, Republic of Korea

**Keywords:** biomimetic underwater robot, longitudinal mode controller design, fish robot model, identification

## Abstract

This paper presents an experimental longitudinal mode control approach for a biomimetic underwater robot. Input–output models for surge velocity and pitch angle were derived through experiments, considering the fish robot body with servo motors and control pins as a single system to solve the problem of fish robots, which are complex and nonlinear, and also contain uncertainty. Closed-loop control systems were designed using PID controllers based on these models, and their performance was verified through simulations and experiments. Surge velocity and pitch angle response models were developed for nominal surge velocities of 0.2 m/s and 0.4 m/s. The surge velocity response models exhibited high agreement rates of 75.25% and 81.23% between the identified linear models and experimental results at 0.2 m/s and 0.4 m/s, respectively. In contrast, the pitch angle response model showed lower agreement rates of 68.02% and 34.24% between the identified linear model and experimental results at 0.2 m/s and 0.4 m/s, respectively. The gain margin and phase margin of the surge controller were 28.7 dB and 116°, and 37.2 dB and 70.6°, respectively. For the pitch response model, the low-frequency gain of the transfer function was very small at −31 dB when the nominal surge velocity was 0.2 m/s; this gain increased to −8 dB when the nominal surge velocity was increased to 0.4 m/s. It was observed that the initial value responses of the pitch angle converged to 0° with some oscillations in both the simulations and experiments. Therefore, it is believed that by identifying a linear model and subsequently designing a controller based on it, the surge velocity of the fish robot can be effectively controlled while stabilizing its pitch angle.

## 1. Introduction

A robotic fish is an underwater platform designed to mimic the locomotion of real fish [1]. Unlike conventional autonomous underwater vehicles (AUVs) that utilize screw propellers [2], robotic fish generate thrust through oscillatory or undulatory fin motions, employing bio-inspired strategies [3]. The primary source of thrust for body and/or caudal fin (BCF) locomotion is the caudal fin motion [1,4], while median and/or paired fin (MPF) locomotion utilizes pectoral fin motions for propulsion [5,6]. In particular, thunniform fish that rely on BCF locomotion are recognized for their high efficiency and swimming speed [7].

A wide range of studies have been conducted on biomimetic fish robots [8,9,10]. Humphreys [11] derived the six-degree-of-freedom (6-DOF) equations of motion for underwater vehicles, including inertia and hydrodynamic forces. The dynamics of the autonomous underwater vehicle Autolycus were later described in detail by Tang [12], providing a practical framework for modeling hydrodynamic effects in real underwater platforms. The UC-Ika 1 tuna-mimetic robot was developed with a four-degree-of-freedom formulation, and its dynamics were validated experimentally at a cruising speed of about 0.29 m/s [13].

Van den Berg et al. [14] introduced the OpenFish platform, a biomimetic robotic fish that employs a wire-driven propulsion mechanism capable of high-frequency tail-beat motions. Yang et al. [15] developed a wire-driven robotic fish based on a double-sine mechanism, demonstrating that cable-driven actuation can effectively reproduce tuna-like body undulation and enhance swimming performance compared to conventional single-sine approaches. Additionally, there are more complex fish models being explored. For example, flying fish with tail-beating glides are under development [16,17], indicating an expansion beyond traditional designs. Most robotic fish utilizing wire-driven mechanisms rely solely on the caudal fin for swimming, while the pectoral and dorsal fins are primarily used for balance [18,19]. Although pectoral fins are mainly employed for stability, they can also facilitate agile maneuvering in robotic fish. For instance, Castano and Tan [6] proposed a dual-loop control method.

Kim et al. [20] proposed an integral sliding mode controller (ISMC) to stabilize an autonomous underwater vehicle (AUV) with modeling errors. Aruna et al. [21] implemented trajectory tracking control for the BAUV using PID, H∞, and feedforward–feedback controllers as representative methods. While most studies have focused on trajectory tracking control, attitude control has also been investigated as an essential factor for vehicle stability. Xiang et al. [22] introduced this aspect by applying a classic PID controller for coordinated 3D path following in underactuated AUVs. Chen et al. [23] developed a 3D-printed biomimetic robotic fish capable of dynamic water quality monitoring in aquaculture. Suebsaiprom and Lin [24] developed a 6-DOF maneuverability model for a carangiform robotic fish using a planar four-link mechanism, highlighting the complexity of capturing trajectory tracking behavior. Barbera, Pi, and Deng [5] developed a dynamic model of a pectoral fin-driven robotic fish and implemented an IMU-based complementary filter to achieve robust attitude estimation and maneuverability. The two-degree-of-freedom (2DOF) PID controller allows for independent tuning of set-point tracking and disturbance rejection [25,26]. Recently, learning-based controllers have also been introduced in soft robotic fish, such as visual learning-based control using super-coiled polymers by Rajendran et al. [27]. Reinforcement learning was applied to a fin-ray effect fish robot by Youssef et al. [28]. Additionally, physics-informed neural networks (PINNs) can be considered a promising approach as they can improve model fidelity and control performance by integrating physics laws with data-driven learning [29].

Gumpina et al. [25] further advanced this field by developing a nonlinear 6-DOF dynamic model of a robotic fish that incorporated hydrodynamic damping, added mass, and restoring forces. Using MATLAB R2024b based system identification tools [30,31], they applied Pseudo-Random Binary Sequence (PRBS) inputs to the nonlinear model and derived linear transfer functions for surge, pitch, and yaw motions. Based on these identified models, they designed a two-degree-of-freedom (2-DOF) PID controller that achieved large gain and phase margins, ensuring robust performance across various operating conditions.

Previous studies have primarily focused on theoretical model-based simulations with known parameters [11,12,25] or open-loop direction and velocity control using RC controllers [32]. But fish robots typically undergo significant design changes during the process of adjusting factors such as buoyancy and the center of gravity. Therefore, while designing a controller based on the original model may theoretically work well, actual experiments often result in poor control [33]. Furthermore, the process of generating torque for pitch angle control from the pectoral fin servo motor and generating thrust from the caudal fin flapping requires highly complex, nonlinear dynamic analysis [19,34]. Therefore, incorporating the dynamics of the pectoral and caudal fins into the six-degree-of-freedom body dynamics results in an overly complex equation, making it difficult to gain insight into controller design and analysis [34].

In this study, to address the problem of fish robots, which are highly complex, nonlinear, and contain uncertainty, the fish robot body with a servo motor and control pins is considered as one system. Thus, the control inputs are the PWM duty ratios provided to the servo motors, and the outputs are the speed or attitude of the fish robot. With different PWM duty ratios signals, the responses of the fish robot, including the impact of the pec-toral fin drive system on pitch dynamics and the impact of the caudal fin dynamics on surge dynamics, were measured. After that, system identification theory was applied to the measured data to obtain a simple input–output relationship for the actual model. Based on the identified model, a PID controller was designed. In particular, the controller was designed to be robust with a gain margin of more than 10 dB and a phase margin of more than 45 degrees to cope with unmodeled uncertainties [35]. The performance was verified through frequency-domain and time-domain simulations, and the controller was implemented in the control device of an actual fish robot. Experiments confirmed that the performance was in good agreement with the simulation results. Rather than relying on complex nonlinear coupled equations, this method of obtaining a dominant linear relationship between input and output, designing a controller based on this, and verifying its performance through simulation and experiments is expected to be applicable to the de-sign of controllers for various types of fish robots and insect-inspired aerial vehicles.

The remainder of this paper is organized as follows: Section 2 describes the experimental setup and the procedures used to collect input–output data from the robotic fish. Section 3 presents the modeling approach, which includes system identification and the linearization of the longitudinal dynamics, as well as the design of PID controllers and the validation of the identified models. Section 4 provides concluding remarks and outlines directions for future research.

## 2. Equation Motion and Experiment Setup

In this section, we present the three degrees of freedom (3-DOF) equations of motion for the longitudinal mode. We also explain the electronic hardware and experimental setup.

### 2.1. Longitudinal Equation of Motion

To describe the longitudinal dynamics of the robotic fish, the 3-DOF motion related to the longitudinal mode is extracted and organized from the general 6-DOF equations of motion. The longitudinal mode includes surge, heave, and pitch motions. The equations of motion for the longitudinal mode are derived using the Newton–Euler law, incorporating key elements such as mass, added mass, fluid damping force, restoring force due to gravity and buoyancy, and propulsive force. The longitudinal equations of motion are presented in Equations (1)–(3). The detailed description of the equations and the notations can be found in [2].


(1) $$\dot{u}=\left(\frac{1}{\left(m-X_{\dot{u}}\right)}\right)\left(Z_{\dot{w}}wq+Z_{\dot{q}}q^{2}-Y_{\dot{v}}vr-Y_{\dot{r}}r^{2}+X_{u\left|u\right|}u\left|u\right|+\left(W-B\right)\sin\theta \;\;\;\;\;\;+\left(m\left(vr-wq+x_{G}\left(q^{2}+r^{2}\right)-z_{G}\left(pr+\dot{q}\right)\right)\right)\right)+X_{c},$$

(2) $$\dot{w}=\left(\frac{1}{\left(m-W_{\dot{w}}\right)}\right)\left(Z_{\dot{w}}\dot{q}-X_{\dot{u}}uq+Y_{\dot{v}}vp+Y_{\dot{r}}rp+Z_{w\left|w\right|}w\left|w\right|+\left(W-B\right)\cos\theta \cos\phi \;\;\;\;+\left(m\left(uq-vp+z_{G}\left(p^{2}+q^{2}\right)-x_{G}\left(rp-\dot{q}\right)\right)\right)\right)+Z_{c},$$

(3) $$\dot{q}=\left(\frac{1}{(I_{yy}-M_{\dot{q})}}\right)\left(Z_{\dot{q}}\left(\dot{w}-uq\right)-\left(Z_{\dot{w}}-X_{\dot{u}}\right)wu-Y_{\dot{r}}vp+\left(K_{\dot{p}}-N_{\dot{r}}\right)rp+M_{q\left|q\right|}q\left|q\right|\;\;\;\;+M_{w\left|w\right|}w\left|w\right|-\left(z_{G}-z_{b}B\right)\sin\theta -x_{G}\left(W-B\right)\cos\theta \;cos\phi \;\;\;\;\;\;\;\;\;-\left(I_{xx}-I_{zz}\right)rp-m\left(z_{G}\left(\dot{u}-vr+wq-x_{G}\left(\dot{w}-uq+vp\right)\right)\right)\right)+M_{c}.$$

Here, u, v, w denote the surge, sway, heave velocities in the body-fixed coordinate system, respectively, while p, q, r represent the roll, pitch, yaw angular velocities. Φ, θ, Ψ denote roll, pitch and yaw angle, respectively. W and B represent the weight and buoyancy, and ($x_{G},\;z_{G}$), ($x_{B},\;z_{B}$) indicate the positions of the center of gravity and the center of buoyancy, respectively. $X_{u},\;Y_{v},\;Z_{w},\;K_{p},\;M_{q},\;N_{r},\;Y_{r}$ and $Z_{q}$ are added-mass coefficients, whereas $X_{u\left|u\right|},\;Y_{v\left|v\right|},\;Z_{w\left|w\right|},\;M_{q\left|q\right|}$ and $N_{r\left|r\right|}$ denote nonlinear damping coefficients. Finally, $X_{c},\;Z_{c}$ and $M_{c}$ represent the forces and moments generated by the control inputs.

In the water tank experiment of this study, the vertical velocity was observed to be significantly smaller than the forward velocity and pitch motion, allowing it to be excluded as a major influencing factor in the subsequent analysis.

### 2.2. Coordinate and Sensor Data Transformation

**Figure 1:**
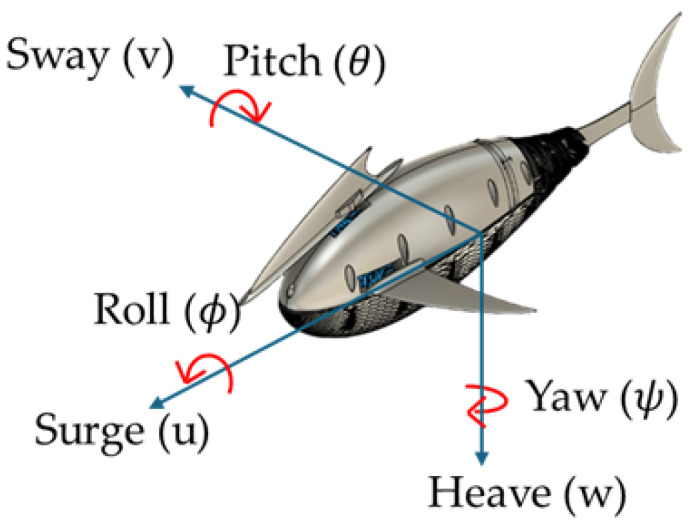
Definition of body frame and variables for longitudinal motion.

The origin of the body-fixed coordinate system is located at the center of buoyancy, as shown in Figure 1. The *x*-axis points forward, the *y*-axis is oriented laterally, and the *z*-axis extends downward. The positions of the center of gravity and the IMU sensor are defined as $\left(x_{G},\;y_{G},\;z_{G}\right)$, ($x_{IMU},y_{IMU},z_{IMU}$), respectively. Euler angles, linear acceleration, and angular velocity are measured from the mounted IMU. Euler angles, linear acceleration, and angular velocity are measured by the mounted IMU.

After the accelerations were measured in the body frame, the bias was first removed, and then the acceleration in the NED frame was obtained by multiplying this by $C_{NB}$ given by Equation (4), where the $C_{NB}$ represents the transformation matrix from body frame-to- NED frame. The Euler angles in (4) are measured by the IMU. After compensating for the gravitational acceleration, the velocity in the NED frame was obtained through integration. The surge velocity in NED frame was calculated using trapezoidal integration of the corrected acceleration over time. In this study, no additional filtering was applied, and the raw data were stored as is to ensure reproducibility.

(4) $$C_{NB}={\left[\begin{array}{ccc} \cos\Psi \cos\theta & \cos\Psi \sin\theta \sin\Phi -\sin\Psi \cos\Phi & \cos\Psi \sin\theta \cos\Phi +\sin\Psi \sin\Phi \\ \sin\Psi \cos\theta & \sin\Psi \sin\theta \sin\Phi +\cos\Psi \cos\Phi & \sin\Psi \sin\theta \cos\Phi -\cos\Psi \sin\Phi \\ -\sin\theta & \cos\theta \sin\Phi & \cos\theta \cos\Phi \end{array}\right]}^{T}$$

Table 1 presents the physical specifications of the underwater robot fish. The robot measures 450 mm in overall length, 90 mm in maximum width, and 160 mm in height. The lengths of the head, body, and tail are 65 mm, 210 mm, and 175 mm, respectively. Its total weight is 2440 g, including additional weights added to ensure neutral buoyancy during the water tank experiments.

### 2.3. Mechanism and Electronic Control System Design

**Figure 2:**
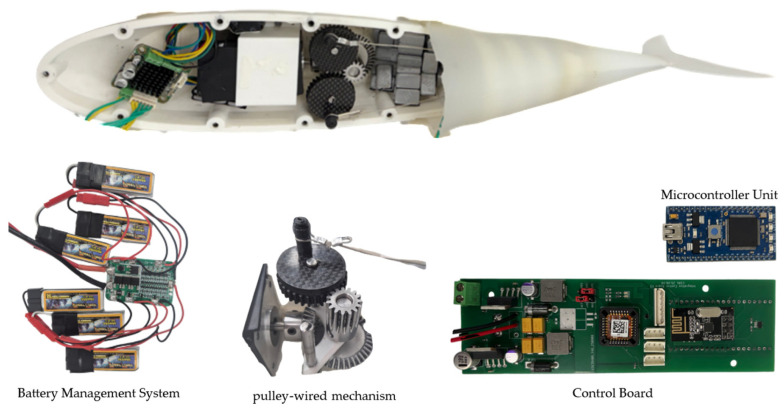
Robot fish pulley-wired mechanism and electronic systems for control.

This robot fish was manufactured using the OpenFish platform developed at Delft University [3], with some structural modifications. Its overall shape features a biomimetic design, including a streamlined exterior and a flexible caudal fin. Propulsion is provided by a BL4270-244064 brushless direct current (BLDC) motor, manufactured by Motorbank Co., Ltd. (Seoul, Republic of Korea), with a rated torque of 0.2 N·m. The motor’s rotation is transmitted to the caudal fin through a pulley system, as shown in Figure 2, causing the fin to oscillate laterally. Screws secure the upper and lower parts of the body, while silicone is used for waterproofing.

An NXP microcontroller unit (MCU) with an ARM Cortex-M3 core, manufactured by NXP Semiconductors N.V. (Eindhoven, the Netherlands), was used for data acquisition and controller implementation. The MTi-3 inertial measurement unit (IMU), manufactured by Xsens Technologies B.V. (Enschede, the Netherlands), measures attitude and linear acceleration. The MTi-3 IMU has an absolute roll and pitch angle error of 0.5° root mean square (RMS). For the gyroscope, the bias instability is $\sigma_{biasIn}^{gyro}=6\;deg/h$ and the noise density is $0.003\;deg/s/\sqrt{H}z$. For the accelerometer, the bias instability is $\sigma_{biasIn}^{acc}=40\;\mu g$ and the noise density is $70\;\mu g/\sqrt{H}z$ [36]. Since the measurement bandwidth is set as 25 Hz, the noise density of the gyroscope and that of accelerometer used in the experiment are $\sigma_{noise\;}^{gyro}=0.015\;deg/s$ and $\sigma_{noise}^{acc}=0.35\;mg$, respectively. Using these values, the surge velocity measurement errors due to random noise and bias instability of the gyroscope and accelerometer are shown in Table 2. The bias instability error, also known as in-run bias stability, represents the extent to which the bias changes over time and corresponds to noise that cannot be compensated [37]. The errors due to bias instabilities are obtained assuming the bias value is fixed during the experiment time.

Furthermore, the IMU is not located at the origin, but at a position r. The acceleration error due to this mounting position is given by Equation (5) [37]. Here, $a_{error}$, α, and ω are the acceleration error, angular acceleration, and angular velocity of the fish robot, respectively.

(5) $$a_{error}=\alpha \times r+\omega \times \left(\omega \times r\right)$$

Considering the actual mounting position and operating time, $r={\left(0,0,-0.006\right)}^{T}$ and T = 7 s. If $\omega ={\left(0,\;10,\;10\right)}^{T}deg/s$ and $\alpha ={\left(0,\;1,\;1\right)}^{T}deg/s^{2}$, for example, the surge velocity error due to the mounting position is approximately −0.00073 m/s, should be added to the errors in Table 2. Since the surge velocity error increases as a function of time, additional velocity compensation devices are required for longer measurement times.

A 24FC512 64-KByte electrically erasable programmable read-only memory (EEPROM), manufactured by Microchip Technology Inc. (Chandler, AZ, USA), was used to store experimental data. This data includes packet counters, Euler angles, acceleration, angular velocity, throttle input, and estimated forward velocity, which are recorded in a EEPROM via I^2^C at a frequency of 50 Hz.

An RF module facilitated external communication, with the RF antenna protruding from the top of the fish robot to ensure consistent communication during the experiment. The caudal fin was driven by a BLDC motor, while S-5086WP waterproof servomotors, manufactured by Hitec RCD Co., Ltd. (Busan, Republic of Korea), controlled the pectoral and dorsal fins. A power board was designed and manufactured to supply power to these electronic devices, utilizing six 3.7 V Lithium Polymer batteries connected in series as the main power source. A Battery Management System (BMS) managed the batteries to extend cell lifespan and ensure stable power delivery. Table 3 illustrates the power distribution of the fish robot. The main power supply drove the BLDC motor for the caudal fin, while 5-volt step-down regulators generated 5 V for the MCU and servomotors. Additionally, a 3.3 V regulator powered the IMU and EEPROM. The motor driver received speed commands from the MCU via Pulse Width Modulation (PWM) and controlled the BLDC motor through three-phase commutation.

The caudal fin is driven by a BLDC motor, and its speed is controlled by the duty ratio of the PWM signal generated by the MCU. The pectoral fins are driven by two synchronized servomotors for pitch control, which are also controlled by the duty ratio of the PWM signal driven by the MCU. The controller controls the velocity and attitude of the fish robot by varying the duty ratio of the PWM signals applied to the BLDC motor and servomotors at a control frequency of 50 Hz. This PWM duty ratio is the input signal used for system identification, and the output is the speed or attitude angle of the fish robot.

For system identification, the PWM duty values driving the BLDC motor and servomotors, along with the speed and attitude values, are stored in EEPROM at a 50 Hz sampling rate. After the experiment, this input/output data is used to derive a model equation between input and output. At this time, the input signal is the duty ratio applied to the BLDC motor or servo motor, so the derived model equation includes the dynamic characteristics of the BLDC motor and drive system, and the servomotors and drive system.

### 2.4. Experimental Setup

**Figure 3:**
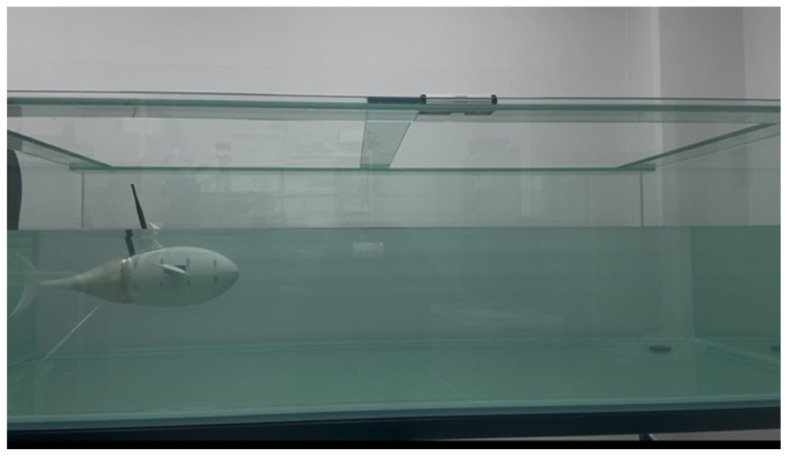
Water tank for the Underwater Fish Robot experiment.

Experiments were conducted in a laboratory water tank with inner-wall dimensions of 1.00 m in width, 2.00 m in length, and 0.60 m in depth (Figure 3). The water tank provided a confined yet controlled environment, enabling repeatable experiments. Within this setup, the underwater fish robot was deployed, and its surge and pitch dynamics were investigated under various actuation inputs. During these tests, velocity, orientation, and angular rate data from the MTi-3 IMU were recorded to the EEPROM for parameter identification and performance evaluation. Video recordings were also made to provide qualitative observations of the swimming behavior and to cross-validate the sensor measurements. The surge direction of the robot was initially aligned with the *x*-axis of the NED frame. The right side of the fish robot was aligned with the *y*-axis, and the bottom was aligned with the *z*-axis of the NED frame.

## 3. Model Identification and Controller Design

For system identification, the excitation signals were set to give high signal when the band limited white noise with noise power of 0.1 was greater than 0, and low signal when it was less than 0. Considering the response time of the fish robot, the sampling time of the white signal was set to approximately 1 s. After fine-tuning to have a constant value like white noise in the main working frequency range of the fish robot, 0 to 10 rad/s, through frequency response analysis, it was used in the actual experiment. The sampling frequency was fixed to 50 Hz during the system identification experiment. A black-box modeling approach was employed for model identification in this paper. In this approach, we selected cases where the fit ratio increased while adjusting the number of zeros and poles. If increasing the order did not further increase the fit ratio, we fixed the order and used the values obtained at that time as the final model values. The roll motion was passively stabilized by positioning the center of gravity (CG) lower than the center of buoyancy (CB). It is assumed that the coupling between small variations in surge velocity and pitch angle is negligible. Model identification experiments were conducted assuming that the two motions operate independently. The longitudinal dynamics are characterized by surge velocity (u), heave velocity (w), pitch rate (q), and pitch angle (θ). This paper focuses on the linear models describing surge velocity and pitch motion, while the heave velocity, which is very small, is not included. The linear models were derived using MATLAB’s identification tool applied to the experimental input–output data. We conducted open-loop experiments by actuating the caudal and pectoral fins via PWM duty ratio variations. From the resulting responses, we identified the linear models between the applied PWM duty signals and the surge velocity/pitch angle that best matched the experimental data. Body angular rates (p, q, r), and Euler angles (roll, pitch, yaw), as well as linear accelerations are measured using an onboard inertial measurement unit (IMU). The linear velocities (surge, sway, heave) are estimated using the IMU data. All signals were saved in EEPROM for parameter identification and performance evaluation.

After model identifications, we designed simple PID controllers to stabilize the surge velocity and pitch angle. The PID controllers we used have the following form (6):

(6) $$C\left(s\right)\;=\;P\;+\;I\cdot \frac{1}{s}\;+\;D\cdot \frac{N}{1+N\frac{1}{s}}.$$

where P, I, and D denote the proportional, integral, and derivative gains, respectively, and N represents the derivative filter coefficient. In the Simulink auto-tuning process, we first examined the open-loop frequency response and set the proportional gain as the initial value to ensure a bandwidth of approximately 1 rad/sec or more. Then, we reviewed both the frequency and step responses via simulation to adjust the integral and derivative gains to ensure appropriate gain and phase margins while maintaining adequate settling time. If performance was unsatisfactory with only the I and D values adjusted, the P value was changed and then the I and D values were adjusted again. For the pitch controller, the integral gain was omitted because it introduces additional phase delays and reduces the stability margin. For practical use, the control loop was designed to meet the following preferred conditions to account for model uncertainties and sensor noise: (1) the gain margin be higher than 10 dB; (2) the phase margin be greater than 45°. The 10 dB gain margin means that stability is maintained even when the gain in the control loop changes by about 3.3 times, and the phase margin of 45 degrees means that stability is maintained even when the phase delay due to the sensor and competition time, etc., becomes up to 45 degrees.

### 3.1. Surge Response and Controller Design

**Figure 4:**
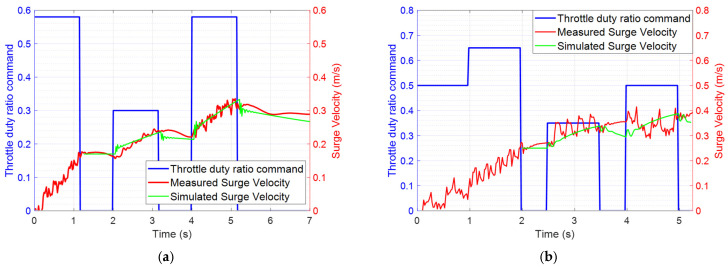
Surge velocity perturbation responses under PRBS input: (**a**) measured surge velocity and linear model response at a nominal surge velocity of 0.2 m/s; (**b**) measured surge velocity and linear model response at a nominal surge velocity of 0.4 m/s.

Surge velocity response was tested at nominal surge velocities of 0.2 m/s and 0.4 m/s. After reaching the nominal velocities, a PRBS input was applied in addition to the nominal command to assess the variation in surge velocity. The input and output variation data were saved to EEPROM and later retrieved for model identification. Figure 4a displays the applied input throttle duty command and the output surge velocity responses at a nominal surge velocity of 0.2 m/s. The blue line represents the throttle duty applied, while the red line shows the surge velocity response. After eliminating the nominal throttle value and surge velocity, we utilized the input and output data to develop linear models using identification tools in MATLAB. The input and output data from 1.2 to 7 s were used for identifying the linear model at a nominal surge velocity of 0.2 m/s. Similarly, we performed the identification for the nominal surge velocity of 0.4 m/s, as shown in Figure 4b, using input and output data from 2 to 5.25 s. The identified linear models for the surge velocity are summarized in Table 4.

**Figure 5:**
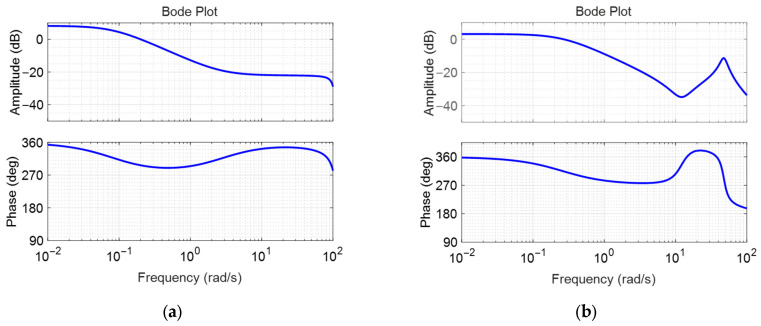
Frequency responses of the identified surge velocity models: (**a**) linear model obtained with nominal surge velocity of 0.2 m/s; (**b**) linear model obtained with nominal surge velocity of 0.4 m/s.

The identified linear models were compared with the measured surge velocity responses to assess identification accuracy. In Figure 4a,b, the measured data and linear model responses yielded fit-to-estimation values of 75.72% at a nominal surge velocity of 0.2 m/s and 81.23% at 0.4 m/s, respectively. Figure 5a,b illustrate the frequency responses of the identified linear transfer functions at nominal surge velocities of 0.2 m/s and 0.4 m/s. As the nominal velocity increased, the bandwidth expanded, leading to faster responses to control inputs. However, the low-frequency gain decreased with increasing nominal surge velocity, indicating that the surge velocity responses become smaller in relation to variations in throttle duty.

PID controllers, as shown in Equation (6), were designed for the model transfer functions in Table 4. Table 5 shows the surge controller parameters obtained using PID tuning.

**Figure 6:**
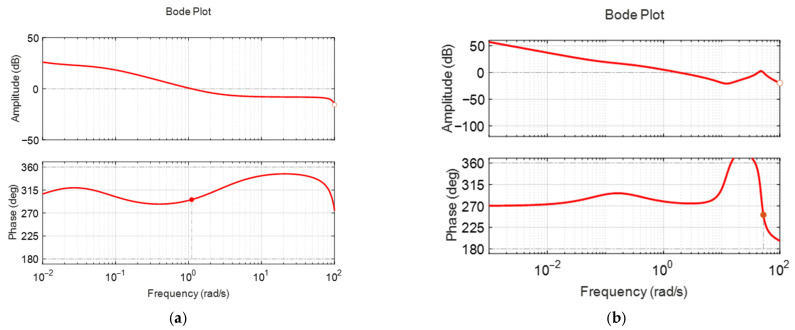
Open loop frequency responses of the identified surge velocity models with feedback controller; (**a**) case with nominal surge velocity 0.2 m/s; (**b**) case with nominal surge velocity 0.4 m/s.

Figure 6a,b display the frequency responses of the surge model with a feedback PID controller, which were used to assess system stability margins. The red solid line represents the frequency response of the system, while the grey dashed line indicates the reference line used for evaluating the stability margins. The bandwidths were 1.1 rad/s and 2.3 rad/s, the phase margins were 116 degrees and 70.6 degrees, and the gain margins were 28.7 dB and 37.2 dB, respectively. The gain margins of 28.7 dB and 37.2 dB mean that the stability of the linear model is maintained even when the gain increases up to 37.2 times and 71.8 times, and the phase margins of 116 degrees and 70.6 degrees mean that the stability is maintained even when the phase delay due to the sensor and controller time delays increases up to 116 degrees and 70.6 degrees, respectively. This is for the case where the model is a linear model, so it does not mean that this stability robustness is guaranteed in the original nonlinear fish robot, but it means that there is a very high possibility that the stability is maintained despite various nonlinear couplings, disturbances, etc., and the actual stability must be verified through experiments. The surge controller exhibited significantly large gain and phase margins. This provided sufficient stability margin but may result in somewhat conservative performance in terms of response time.

**Figure 7:**
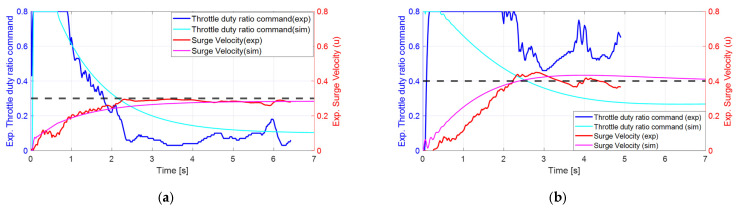
Step responses of the closed-loop surge velocity: (**a**) case with nominal surge velocity 0.2 m/s; (**b**) case with nominal surge velocity 0.4 m/s.

Figure 7 illustrates the surge velocity responses to step commands, comparing simulated results from the linear model with actual experimental data. The black dashed line represents the reference input signal corresponding to the throttle duty ratio command. In Figure 7a, a nominal surge velocity of 0.2 m/s is depicted. The linear model’s response to a 0.3 m/s step command from a stationary state is compared with the experimental results. The simulation indicates a rise time of 3.3 s with no overshoot, while the experiment shows a rise time of approximately 2 s, also exhibiting no overshoot. The controller output initially saturates at an 80% throttle duty ratio and recovers from saturation after about 0.8 s in the simulation and approximately 0.9 s in the experiment. The experimental data acquisition was stopped at around 6.4 s as the fish robot reached the end of the water tank. Overall, the simulation and experimental results show good agreement, despite the small size of the water tank.

Figure 7b illustrates the case where the nominal surge velocity is 0.4 m/s. It compares the response of the linear model with experimental results following a step command of 0.4 m/s from a standstill. In the simulation, the rise time is 2.0 s, with an overshoot of 7.5%. In contrast, the experiment shows a rise time of 1.7 s and an overshoot of approximately 10%, which is comparable to the simulation results. Initially, the controller output saturated at a throttle duty ratio of 80%, recovering from saturation after about 0.4 s in the simulation and approximately 2.0 s in the experiment. The experimental data is truncated at around 4.9 s because the fish robot reaches the end of the water tank. The saturation time of the control input differs from the simulation results, likely due to the plant starting at 0 m/s, significantly lower than the nominal velocity of 0.4 m/s, creating a substantial error gap between the initial linear model and the plant. Additionally, due to nonlinear coupling, the experimental velocity response exhibits slight fluctuations even after reaching the target value of 0.4 m/s. However, overall, the velocity response profiles remain consistent with each other.

**Figure 8:**
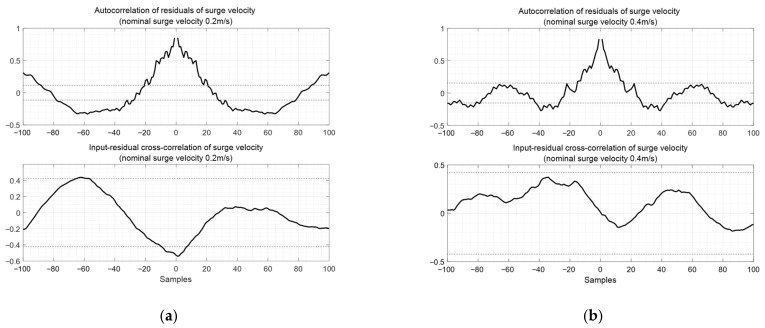
Autocorrelation of residuals of surge velocity and input-residual cross-correlation of surge velocity: (**a**) case with nominal surge velocity 0.2 m/s; (**b**) case with nominal surge velocity 0.4 m/s.

Figure 8 shows the cross-correlation of residuals of surge velocity and the input-residual cross-correlation of surge velocity, showing that the modeling error is relatively large when the reference speed is low. The black dashed line represents the 95% confidence level for both the autocorrelation and cross–correlation of the residuals.

Table 6 shows the root mean square error (RMSE) between the surge velocity model predictions and experimental data. The absolute error is larger when the nominal surge velocity is 0.4 m/s than when it is 0.2 m/s, which is partly due to the larger absolute moving speed.

### 3.2. Pitch Response and Controller Design

**Figure 9:**
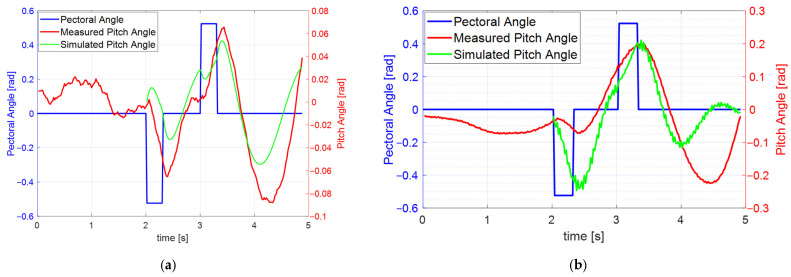
Pitch angle responses to the PRBS input of pectoral fin angles: (**a**) Measured pitch angle and simulated response from the identified model at a nominal surge velocity of 0.2 m/s; (**b**) Measured pitch angle and simulated response from the identified model at a nominal surge velocity of 0.4 m/s.

To obtain the pitch response, a PRBS of ±0.5 radians was applied to the pectoral fins angle servo input, and open-loop tests were conducted to collect input–output data. We performed experiments at nominal surge velocities of 0.2 m/s and 0.4 m/s. After reaching the nominal velocity, the PRBS input was applied to assess the variation in the pitch angle. The input and output variation data were saved to EEPROM for model identification. Figure 9a,b illustrate the pectoral fins angle input and the pitch angle response at nominal surge velocities of 0.2 m/s and 0.4 m/s, respectively. The blue line represents the input, while the red line indicates the measured pitch angles in radians. Approximately 2 s were required to reach the nominal surge velocity. Using the input and output data collected after this period, a linear model was developed with the MATLAB System Identification Tool. The green lines represent the responses of the identified pitch dynamic models. The fit-to-estimation values were 68.02% at 0.2 m/s and 34.24% at 0.4 m/s. The reason for the low estimated fit of the model obtained at the nominal frequency of 0.4 m/s is the short measurement time and the influence of the generated surface waves on the attitude due to the small size of the water tank. Referring to Equation (3), we can see that the pitch dynamics include nonlinear coupling terms, including the pitch rate q and heave velocity w, which are coupled with the surge velocity u. These appear to have reduced the accuracy when approximating the input–output relationship as a linear system. The pitch input generates heave velocity w, which, when multiplied by the surge velocity u, acts as a disturbance. The identified linear transfer functions are summarized in Table 7.

**Figure 10:**
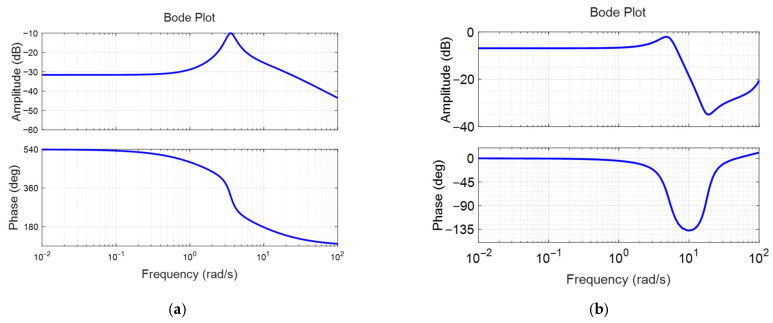
The open-loop frequency responses of the identified pitch models: (**a**) case with nominal surge velocity 0.2 m/s; (**b**) case with nominal surge velocity 0.4 m/s.

Figure 10a,b illustrate the frequency responses of the identified pitch angle linear transfer functions at nominal surge velocities of 0.2 m/s and 0.4 m/s, respectively. As the nominal surge velocity increases, the bandwidth also increases, leading to a faster pitch response for the pectoral fin input. Additionally, the low-frequency gain rises, resulting in a larger pitch angle for a given pectoral fin input. However, the overall gain in the pitch angle response remains quite small. Specifically, at a nominal surge velocity of 0.2 m/s, the low-frequency gain is approximately −31 dB, indicating that the pitch moment generated by the pectoral fin is minimal due to its proportionality to the square of the surge velocity. Consequently, the pitch moment at a surge velocity of 0.2 m/s is very small. The resonance point occurs at an angular velocity of 3.6 rad/s, where the damping coefficient is very low at 0.14. Relatively large motion is expected when frequencies are applied near this point. At a nominal velocity of 0.4 m/s, the low-frequency gain and resonant frequency are approximately −8 dB and 5.27 rad/s, respectively. At this time, the damping coefficient is also low as 0.279. The gain in the low-frequency range is significantly greater than that at 0.2 m/s, which is expected to enhance the pitch motion stabilization.

Simple PD controllers, as shown in Equation (6), were designed for the model transfer functions listed in Table 7. Table 8 displays the pitch controller parameters obtained through PD tuning.

**Figure 11:**
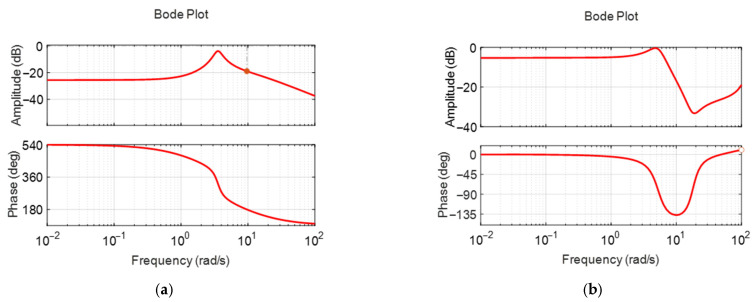
Open loop frequency responses of the identified pitch models with feedback controller: (**a**) case with nominal surge velocity of 0.2 m/s; (**b**) case with nominal surge velocity of 0.4 m/s.

Figure 11a,b display the frequency responses of the identified pitch plant and controller at frequencies of 0.2 m/s and 0.4 m/s, respectively. When the nominal surge velocities are 0.2 m/s and 0.4 m/s, the gain margin is 20 dB and infinity, and the phase margin is infinity and 65 degrees, respectively. As in surge velocity control, very large gain and phase margins are observed. However, this does not guarantee the stability of the nonlinear fish robot to this extent. It means that the designed controllers are very likely to maintain stability despite the nonlinear coupling and disturbances included in the actual fish robot. Actual stability must be verified through experiments.

**Figure 12:**
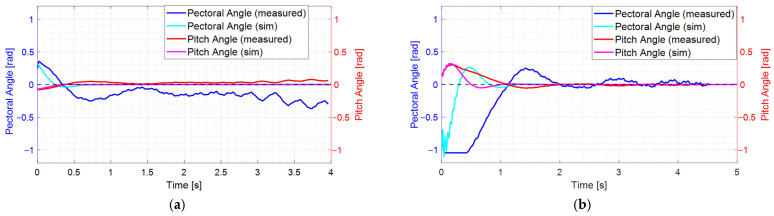
Pitch responses to initial values: (**a**) case with nominal surge velocity 0.2 m/s; (**b**) case with nominal surge velocity 0.4 m/s.

Figure 12 demonstrates that, given a non-zero initial attitude, the PD controller converges to the reference pitch attitude of 0 rad. The grey dashed line indicates the zero-reference angle. Figure 12a presents the pitch controller response at a nominal surge velocity is 0.2 m/s. Simulation and experimental results are compared together. The experimental results are similar to the simulation results, showing that the pitch angle converges to 0 rad over time. Figure 12b shows the pitch controller response when the nominal surge velocity is 0.4 m/s. Both experimental and simulation results reveal that the pitch angle converges to 0 rad, albeit with transient oscillations. As the pectoral pin angle increases, nonlinearity also increases. Furthermore, the controller is initially saturated. Consequently, the experimental results for pitch response converge to 0 rad more slowly than the simulation results.

**Figure 13:**
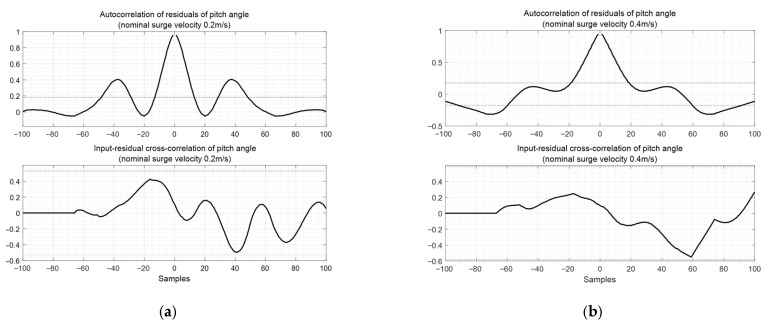
Autocorrelation of residuals of pitch angle and input-residual cross-correlation of pitch angle: (**a**) case with nominal surge velocity 0.2 m/s; (**b**) case with nominal surge velocity 0.4 m/s.

Figure 13 shows the autocorrelation of residuals of surge velocity and the input-residual cross-correlation of surge velocity. The autocorrelation exhibits characteristics that differ from white noise, indicating room for improvement. The black dashed line represents the 95% confidence level for both the autocorrelation and cross–correlation of the residuals.

Table 9 shows the RMSE between the pitch model’s predictions and experimental data. The absolute error is larger when the nominal surge velocity is 0.4 m/s than when it is 0.2 m/s, which is partly due to the larger absolute initial angle.

## 4. Conclusions and Future Works

The longitudinal mode input–output transfer function of a biomimetic fish robot was derived through experiments. Input–output measurements were taken while applying PRBS random commands, and this input–output data was used to establish the transfer function. The surge velocity and pitch responses were obtained at nominal surge velocities of 0.2 m/s and 0.4 m/s. A PID controller was implemented for surge control, while a PD controller was used for pitch control.

The surge velocity response model produced excellent fit-to-estimation values of 75.72% and 81.23% for nominal surge velocities of 0.2 m/s and 0.4 m/s, respectively, even though the tank size was small. The surge velocity controller demonstrated strong compliance with the reference velocity command, with experimental and simulation results closely matched. When a 0.4 m/s surge velocity command was applied, the drive output initially saturated, resulting in slightly slower velocity response time.

The pitch response model yielded fit-to-estimation values of 68.02% and 34.24% for nominal surge velocities of 0.2 m/s and 0.4 m/s, respectively. Although the estimated fit-to-estimation at 0.4 m/s was low, the controller based on it demonstrated good performance. At a nominal surge velocity of 0.2 m/s, the transfer function gain at low frequencies was very small, approximately −31 dB. In contrast, at a nominal surge velocity of 0.4 m/s, the low-frequency gain increased to −8 dB, along with an increase in bandwidth. Using a PD controller, the response to the initial pitch angle was compared through experiments and simulations. Both experiments showed that the pitch angle soon converged to zero and stabilized.

Future research topics include improving model accuracy, controller robustness, and extending the application to other types of fish robots or those operating at different speeds. To improve model accuracy, research is needed on system identification techniques, including regularization, and on the application of nonlinear identification methods, such as the NARX model and neural networks. Furthermore, alternative modeling approaches, including gray-box models and subspace identification, are needed to improve model estimation accuracy. On the control side, advanced strategies, such as model predictive control (MPC) and adaptive controllers, are needed to achieve more accurate and stable responses. While the current controller is designed for fixed-speed operation, gain scheduling techniques could be introduced to automatically adjust controller gains based on various surge speeds and operating conditions. Furthermore, because the control force generated by the pectoral fins is proportional to the square of the forward speed, future research should examine how the low-frequency gains of the pitch model vary under different operating conditions. Furthermore, sensitivity analysis [38] is necessary to evaluate the impact of changes in key parameters and surge rates on the robustness of the model and controller. Such quantitative analysis can identify the most critical parameters for performance and help improve future control designs.

The framework proposed in this study is expected to be effective in obtaining input–output relationships for the entire system, especially when the dynamics of the actuators and body are complex or nonlinear. In particular, it is expected to be useful in identifying models for speed control of wing-flapping bird or insect-inspired aerial vehicles, in addition to fish robots, and designing controllers based on these models.

## Figures and Tables

**Table 1:** Physical specifications of robot fish components.

Components	Value	Unit
Overall length of the robotic fish	450 × 90 × 160 (Length × Width × Height)	mm
Head length	65	mm
Body length	210	mm
Tail length	175	mm
Total weight	2440	g
Center of buoyancy	(0.0, 0.0, 0.0)	mm
Center of gravity	(8.6, 0.0, 2.4)	mm
Coordinate of IMU	(0.0, 0.0, −6.0)	mm

**Table 2:** Estimated surge velocity errors due to IMU measurement errors when T = 7 s.

Error Sources	Surge Velocity Error Standard Derivation	Values (m/s)
Accelerometer bias instability	$v_{biasIn}^{acc}=\sigma_{biasIn}^{acc}\times T$	0.003
Accelerometer noise	$v_{noise}^{acc}=\sigma_{noise}^{acc}\times \sqrt{T}$	0.009
Gyroscope bias instability	$v_{biasIn}^{gyro}=\frac{1}{2}\times g\times \sigma_{biasIn}^{gyro}\times T^{2}$	0.007
Gyroscope noise	$v_{noise}^{gyro}=\frac{1}{\sqrt{3}}\times g\times \sigma_{noise}^{gyro}\times T^{1.5}$	0.028

**Table 3:** Power distribution of the electrical system.

Subsystem	Voltage Range (V)	Remarks
Motors and motor drivers	22.2–25.2	Directly from batteries
Servomotors	5	Two 5 V regulators
MCU and Buffer	5	Single 5 V regulator
IMU, EEPROM	3.3	Single 3.3 V regulator

**Table 4:** Identified surge velocity linear transfer functions with different nominal surge velocities.

Nominal Caudal Frequency (Hz)	Nominal Surge Velocity	Identified Linear Transfer Functions
2.00	0.2 m/s	$\frac{33.34\;s^{3}+25.12\;s^{2}+394,100\;s+1,081,000}{s^{4}+{441.3\;s}^{3}+25,840\;s^{2}+5,001,000\;s+420,600}$
4.00	0.4 m/s	$\frac{10.06\;s^{2}-34.52\;s+161.7}{s^{4}+{7.421\;s}^{3}+28.99\;s^{2}+180.4\;s+113.7\;}$

**Table 5:** Parameters of continuous PID surge controllers.

Model	P	I	D	N
Condition 1	5.0	0.05	−0.5	20.0
Condition 2	1.55	0.62	−0.34	25.0

**Table 6:** Differences between simulation and experimental results of open-loop and closed-loop surge responses at 0.2 m/s and 0.4 m/s.

Model	Open Loop (m/s, RMS)	Closed Loop (m/s, RMS)
Nominal velocity 0.2 m/s Surge	0.014	0.106
Nominal velocity 0.4 m/s Surge	0.026	0.045

**Table 7:** Identified pitch angle linear transfer functions with different nominal surge velocities.

Nominal Caudal Frequency (Hz)	Forward Suge Velocity	Identified Linear Transfer Functions
2.00	Condition 1 (0.2 m/s)	$\frac{-0.6675\;s^{2}+3.792\;s-3.894}{s^{3}+12.81\;s^{2}+24.7s+148.5\;}$
4.00	Condition 2 (0.4 m/s)	$\frac{1.765s^{3}+681.4\;s^{2}+5741\;s+216,200}{s^{4}+4.294s^{3}+17,200\;s^{2}+50,440\;s+477,200\;}$

**Table 8:** Parameters of PD pitch controllers, including the derivative filter coefficient N.

Model	P	D	N
Condition 1	2.0	0.5	20
Condition 2	1.2	0.3	20

**Table 9:** Differences between simulation and experimental results of open-loop and closed-loop pitch responses at 0.2 m/s and 0.4 m/s.

Model	Open Loop (rad, RMS)	Closed Loop (rad, RMS)
Nominal velocity 0.2 m/s Pitch	0.037	0.043
Nominal velocity 0.4 m/s Pitch	0.111	0.111

## Data Availability

The experimental data supporting the findings of this study are publicly available in the Zenodo repository at DOI: 10.5281/zenodo.17096646.
